# Navigating the disease landscape: knowledge representations for contextualizing molecular signatures

**DOI:** 10.1093/bib/bby025

**Published:** 2018-04-19

**Authors:** Mansoor Saqi, Artem Lysenko, Yi-Ke Guo, Tatsuhiko Tsunoda, Charles Auffray

**Affiliations:** 1 Mansoor Saqi Data Science Institute, Imperial College London, UK; 2 Artem Lysenko Laboratory for Medical Science Mathematics, RIKEN Center for Integrative Medical Sciences, Yokohama, Japan; 3 Yi-Ke Guo Data Science Institute, Imperial College London, UK; 4 Tatsuhiko Tsunoda Laboratory for Medical Science Mathematics, RIKEN Center for Integrative Medical Sciences, Yokohama, Japan CREST, JST, Tokyo, Japan Department of Medical Science Mathematics, Medical Research Institute, Tokyo Medical and Dental University, Tokyo, Japan; 5 Charles Auffray European Institute for Systems Biology and Medicine, Lyon, France

**Keywords:** precision medicine, molecular medicine, multi-omics, disease modeling, integrated knowledge networks

## Abstract

Large amounts of data emerging from experiments in molecular medicine are leading to the identification of molecular signatures associated with disease subtypes. The contextualization of these patterns is important for obtaining mechanistic insight into the aberrant processes associated with a disease, and this typically involves the integration of multiple heterogeneous types of data. In this review, we discuss knowledge representations that can be useful to explore the biological context of molecular signatures, in particular three main approaches, namely, pathway mapping approaches, molecular network centric approaches and approaches that represent biological statements as knowledge graphs. We discuss the utility of each of these paradigms, illustrate how they can be leveraged with selected practical examples and identify ongoing challenges for this field of research.

## Introduction

Owing to technological advances allowing rapid and low-cost profiling of biological systems, multiple types of omics data are now routinely collected from patient cohorts in studies of human diseases. These data can lead to a new taxonomy of disease [1]. Diseases that were previously considered to be single homogeneous conditions may in fact be collections of several disease subtypes. Identification of subtypes allows targeting of the underlying molecular processes involved in the particular form of disease associated with the subtype, and can lead to more personalized therapeutic strategies. A major challenge for translational medicine informatics is the effective exploitation of these data types to develop a more complete picture of the disease, in particular a description of how changes at the molecular level are associated with the disease mechanism and disease pathophysiology. The molecular profiles by themselves do not, in general, offer immediate insight into the mechanism of disease or the underlying causes, and may be of limited utility for suggesting targets for therapeutic intervention. Putting these molecular patterns into a broader biological context represents a useful approach for understanding the underlying themes involved in the disease pathology, and this involves integrating the molecular profiles with other data types, including pathways, cellular and physiological data.

Together with data warehousing and data analytics, the contextualization of data emerging from high-throughput experiments is an important component of a translational informatics pipeline. The contextualization of experimental data is facilitated by mapping the data to background knowledge, which can include information at multiple levels of granularity. An effective representation of the disease needs to relate disease-specific information to background knowledge so as to help researchers identify how dysfunctional proteins, pathways or other molecular processes lead to the cellular or physiological changes contributing to its aetiology.

Here, we review efforts to represent the context of disease-implicated genes, and we suggest that they can be divided into three broad themes, namely, pathway-centric, molecular network centric and approaches that represent biological statements as a knowledge graph (Table 1). We describe the advantages and drawbacks of the different representations. We do not discuss the details by which the genes are mapped to pathways or networks (for review of approaches to data interpretation, see for example [2]).

**Table 1:** Approaches to knowledge representation for contextualizing disease biomarkers

Approach	Examples of Formats/Frameworks	Advantages	Drawbacks
Pathway- centric	SBML	Ease of Navigation (e.g. using NaviCell, Google Maps API)	Difficult to represent disease context
	SBGN		
	BioPax		
Integrated molecular networks	GeneMania	Easy-to-use resources and tools	Difficult to represent disease context, although connecting layers of information can provide some context
	STRING		
Knowledge graphs	openBEL	Agility of graph databases; semantic web approaches offer a federated solution; openBEL framework captures context	Lack of formal ontology in graph database representations; Semantic Web solutions do have a formal ontology but lack agility of graph databases such as Neo4j
	RDF		
	Malacards		
	BioXM^TM^		

## Difficulties with representations of disease mechanism

The aim of contextualization is typically to obtain insight into disease mechanism. However, defining disease mechanism is not straightforward. The most appropriate representation of the context depends to some extent on what data are available and what questions are being asked. Disease mechanisms can be represented at different levels of granularity. At high granularity, a disease mechanism would describe all known temporal steps, for example the detailed steps in a signalling pathways, so that the downstream consequences of proteins, aberrant in the disease condition, can be followed. However, information about the disease at lower resolution, for example describing only indirect relationships or correlative relationships, can also give mechanistic insight. Examples of such low-level granularity descriptions of disease include statements like: *STAT6* activation is linked to mucous metaplasia, or Fluticasone up-regulates expression of *FKBP51* in asthmatics (see [3]) or gene *ADAM33* implicated in asthma is also implicated in chronic obstructive pulmonary disease and essential hypertension [4].

Hofmann-Apitius [5] describes mechanism as a causal relationship graph that involves multiple levels of biological organization [5]. Recently, in the Big Mechanism Project (BMP) [6] efforts have been initiated toward building mechanistic models of large, complex biological systems such as those involved in cancer, using large-scale text mining (TM) followed by model construction using a variety of frameworks. Cohen [6] describes mechanism in terms of a model *M*, which maps an input *x* to an output y, *y: M: f^(x)(ax+ε)* => y where the real mechanism *f* is approximated by f^. An important part of the goal of the BMP is not only to develop a collection of such models but also to automate the process of their construction. Therefore, a large part of the project’s efforts is devoted to development of sophisticated TM systems that can automatically capture such mechanistic models directly from the literature [6]. The conceptualization of a mechanistic model put forward by Cohen [6] implies that such a model would be made of parts that reflect some corresponding real components. This view provides a natural way of combining the models (as such parts can be mechanistic models themselves), though it also follows that at some level a simplification will need to be made, e.g. for understanding the regulatory cascade, it may not really be desirable to model the process at the level of individual atoms of the protein molecules involved. Potentially, in the scientific literature, such an abstraction may be set at different levels, as human disease may be, for example, studied at physiological and molecular levels with causal mechanistic relationships being found at both of them. One of the ongoing challenges in achieving the mechanistic understanding of disease is to develop tools and formalisms that can capture, model and reconcile the representations of these different perspectives.

There is much information that may help to suggest disease mechanisms to be included in an integrated representation of disease. For example, information from disease-associated single-nucleotide polymorphisms (SNPs), information from protein–protein interactions and sequence similarity, information on the effects profiled in mutational studies in model organisms and the relationships of molecular variations with physiological and cellular changes. The suitable representation of this information in a manner that can be efficiently stored, envisioned and queried is important for obtaining a mechanistic understanding of disease.

**Figure 1:**
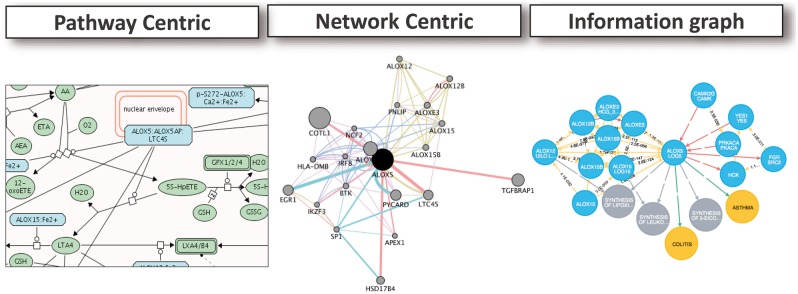
Examples of representations for contextualizing disease associated genes. A pathway centric view of the network neighbourhood of ALOX5 from ReactomeViz, using the Cytoscape plug-in (left). A molecular network centric view using the GenMania Cytoscape plug-in (middle). A heterogeneous network, including proteins, pathways and diseases, constructed and displayed using Neo4j (right).

What is the most appropriate way to represent disease-related information in a computable format that can be useful for hypothesis generation and for obtaining mechanistic insight? To some extent, this depends on what questions are being asked of the data. Here, we describe three approaches to representation of contextual information, namely, pathway-centric approaches, molecular network centric approaches and information graphs (Figure 1). We contend that data integration is a key component of all three approaches.

Network and graph-based representations are prodigiously effective representations for integration, query, exchange and visualization of numerous possible relationships and interactions between biological entities. Owing to well-studied formal mathematics of graphs, they also often serve as a key data structure for algorithmic, machine learning and statistical approaches for analysis of these data. Construction of biological networks from high-throughput experimental data is an essential first step that enables many types of analysis discussed below. While we recognize importance of this research, in this review, our focus will be primarily on downstream applications of biological and knowledge networks for molecular signature contextualization, but for completeness, we would like to recommend the following works, which cover this closely related topic in detail [7–10]. For convenience of readers, we have included a list of abbreviations used throughout the article in Table 2.

**Table 2:** List of abbreviations

Abbreviation	Expanded name	Comment
API	Application programming interface	A set interfaces, tools and functions used for creation of applications
BEL	Biological Expression Language	A curation language for structured capturing of data about biological systems and experiments
BELIEF	BEL Information Extraction workFlow system	A framework for automated parsing of information into BEL format
BioPAX	Biological Pathways Exchange language	A standard that formally defines biological pathway conceptualization in OWL format
BMP	Big Mechanism Project	A project by US Department of Defense for automated construction of mechanistic cancer models from scientific literature
COPD	Chronic Obstructive Pulmonary Disease	A lung disease characterized by impeded breathing and phenotypically similar to asthma
Cypher		A query language for Neo4J graph database
DBMS	Database management system	A software providing capabilities to mediate storage, query and manipulation of data
EBI	European Bioinformatics Institute	
EFO	Experimental Factor Ontology	
GO	Gene Ontology	One of the most widely used ontologies for representing functions of biological entities
GWAS	Genome-wide association study	An observational study that relates germ-line variations between individual to phenotypes
IR	Information Retrieval	A process of extraction of relevant information from some wider superset
Kappa		A rule-based, declarative language used mostly in molecular biology domain
Neo4j		Graph-based database solution
OBO	Open Biomedical Ontologies initiative	OBO format is alternative language for authoring ontologies; mainly used in biological sciences
OWL	Ontology Web Language	Currently most widely used language for authoring ontologies
PD	Process Description language	An extension of SBGN standard with additional capabilities to represent temporal aspects of biological processes
PPI	Protein-protein interaction	
RDF	Resource Description Format	Core data exchange format for the Semantic Web
SBGN	Systems Biology Graphical Notation	A standard for graphical representation for biological and biochemical domain
SBML	Systems Biology Markup Language	An XML-based format for storing and exchanging biological models
SBO	Systems Biology Ontology	
SNP	Single Nucleotide Polymorphism	A single-base variant or mutation in a genomic sequence
SQL	Structured Query Language	Family of similar query languages used for data management in relational databases
TM	Text Mining	A process of extraction of structured data from free text
TMO	Translational Medicine Ontology	
URL	Uniform Resource Locator	Web address, resolving to a resource on the Web
XML	Extensible Markup Language	Popular meta-language for document markup

To provide a practical illustration of how different types of contextualization approaches can be used, we have included examples based on a severe asthma differential expression gene signature and a manually curated core asthma gene signature acquired from DisGeNET database [11]. The differential expression signature was computed from the study by Voraphani and Gladwin [12] (GSE43696), which profiled transcriptomics of moderate and severe asthma as well as healthy controls. A full description of all analysis performed is outlined in detail in the Supplementary Methods. These examples were developed purely to illustrate the approaches discussed in this review, and potentially other examples relevant to asthma could have been chosen.

## Contextualization using integrated networks

Networks or graphs are sets of nodes and edges where a node represents a concept and an edge represents a relationship. Biological concepts can correspond to real, physical entities like molecules, genes and proteins or represent more abstract entities, like typed groups (pathways, functional categories) and diseases. The relationships represent a meaningful association between these concepts, such as ‘protein A interacts with protein B’, ‘enzyme participates in pathway’ or ‘protein is a transcription factor’. In many network studies in systems medicine, the nodes are restricted to particular single type (such as protein), and such graphs are termed homogeneous. In this interpretation, an edge can represent a single relationship type such as a protein–protein interaction, or it can represent multiple types of evidence that associates the two protein nodes. For example, in the STRING protein–protein interaction (PPI) database [13], evidence types from multiple sources are used to suggest functional associations between proteins. The evidence types include genome location, co-occurrence in the scientific literature and reported associations in other databases. Mechanistic insight into diseases can be obtained by mapping disease-associated genes (e.g. identified from omics data analyses) to such networks. The network neighbourhood of the disease genes provides a context and can suggest new candidates as well as common themes at the level of molecular processes and pathways that may be associated with disease pathogenesis. GeneMania [14] is a Cytoscape App that enables the construction of network neighbourhoods from a set of seed genes by integration of a number of constituent networks (e.g. PPI, shared protein domains, co-expression).

Network representation can be leveraged to discover high-level patterns that underlie organization of complex biological systems, and more specifically identify patterns relating to development of disease. One prominent early example of this was from the work of Barabasi and co-workers [15], which represented disease–gene relationships as a bipartite graph from which monopartite disease–disease and gene–gene graphs could be obtained (the human–disease network and the disease–gene network) giving information about disease comorbidities and thereby contextualising known disease genes. By combining the disease–gene network with known PPI networks, it was demonstrated that the corresponding disease-associated proteins for a given disease have a greater tendency to interact in a PPI network. Likewise, proximity of genes in the network can be a strong indicator of their involvement in similar processes. Genes associated with a given disease tend to be closer together in the interactome, and overlapping disease neighbourhoods are related to greater comorbidity [16].

The network can also be analysed to identify notable topological features, many of which have been associated with biologically meaningful properties. One of such features is the modular organization of the network. Modules are groups of nodes that are more interconnected to other nodes within the region, than to other nodes outside the region [17]. In PPI networks, modules have been shown to correspond to functionally similar groups of proteins, which in some cases may be relevant to disease processes. One such example was identified by [18], who, using genes from seven stress-related diseases together with the STRING network, identified a common subnetwork that may provide insights into comorbidity of these diseases. An early example is the network model for asthma [19] that combined information from gene co-expression taken from five public gene expression studies, with PPI networks, and information from annotation sources. The resulting ‘global map’ was then used to explore common themes in asthma pathogenesis.

A powerful example of exploiting the network neighbourhood of known disease genes to get mechanistic insight is given in [20]. Using a set of 129 asthma implicated (‘seed’) genes that map to the human interactome together with a novel algorithm [16] for module detection, Sharma *etal.* [20] identified a potential asthma disease module containing 441 genes including 91 seeds. A large number (162) of pathways had at least half of their genes in the putative disease module. However, a strategy involving prioritizing genes on the basis of their asthma relevance using gene expression and *GWAS* information enabled a ranking of pathways and subsequent identification of pathways, not traditionally associated with asthma. The plausibility of one of these (the *GAB1* signalosome) was shown experimentally. The authors also explored the differential expression of the *GAB1* gene in other immune-related diseases. This study illustrates the use of both pathways and networks with other supporting evidence (e.g. gene expression, GWAS) to contextualize disease genes and provide new avenues for understanding disease mechanisms. It highlights the need for knowledge representations that can capture multiple levels of information such as the conditions in which a gene may be up-regulated.

One approach to representation of the complex networks that are associated with disease is to decompose the information into layers, with each layer representing information at different levels of biological organization. Radonjic and co-workers [21, 22] use network-based methods to represent and integrate information at three levels, namely, differentially expressed biological processes, transcription factors whose target proteins are differentially expressed and physiological information such as energy intake and weight. They explored adaptation of white adipose tissue to a high-fat diet, a biological system that may give clues as to which molecular processes are involved in obesity and type 2 diabetes. Gustafsson *et al.* [17] discussed the concept of multilayer disease modules as a possible framework to generate predictive markers that capture multiple types of disease-related information (PPI, symptoms, etc.). The integration of the layers and the inclusion of addition contextual information associated with the nodes and relationships of the network (e.g. cell-type specificities) are important for mechanistic insight into disease. Network-based data fusion approaches to integration of multiple types of biological information relevant to disease are described by [23, 24], and these offer the potential to predict new disease-associated genes as well as to explore disease–disease relationships.

The network-based integration of different types of information can suggest underlying mechanisms of disease. Huan and co-workers [25] perform an integrated analysis to explore mechanisms of blood pressure regulations. They used weighted gene co-expression network analysis (WGCNA) [26] to identify co-expressed modules that showed correlation to blood pressure measurements, and integrated SNP data as well as PPI networks and used Bayesian analysis to identify key drivers of the biological process, one of which was selected for further experimental study. The identification of associations between diseases can suggest a common mechanistic basis [27] and can be used predictively [28]. Molecular network approaches to representing disease landscapes offer particular views rather than a unified framework. In some ways, they are similar to information overlay methods in data analytics. Points of correspondence between the different views (networks) need to be found, to get an integrated picture that will be useful for suggesting mechanism.

**Figure 2:**
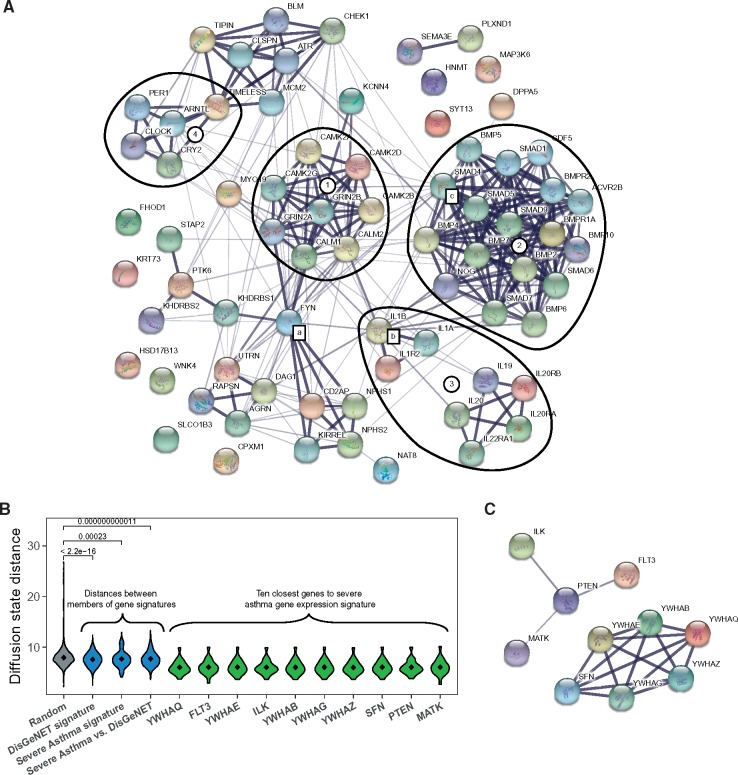
Analysis of severe asthma differential expression gene signature in the context of STRING protein association network. (**A**) Fifty genes in the first shell of the 22-gene signature; (a–c) high-degree module interconnectivity genes; (1) calmodulins and *HIF-1* signalling pathway, (2) *TGF-beta* signalling pathway, (3) cytokine–cytokine receptor interaction pathway and (4) circadian rhythm-related genes. (**B**) Diffusion state distance of gene sets; this measure is derived from similarity of random walk profiles for each pair of nodes. In grey—random pairs of nodes. Distances between members of asthma signatures to each other are shown in blue (severe asthma versus DisGeNet category shows distances between members of the two sets). Finally, 10 closest genes to the differential expression signature set are shown in green. (**C**) Ten closest genes to the severe asthma signature visualized in the STRING database viewer.

To illustrate these contextualization strategies, 22 genes from the differential expression signature (derived from GSE43696 study as outlined in Supplementary Methods) were mapped to protein association network from STRING database. First, the results were explored using Web-based interactive visualization offered by STRING database (Figure 2). As proteins realize their function through complex sets of interactions, implications of gene expression changes can often be better understood by assessing its impact on the wider interactome. The top panel shows disease signature genes as well as 50 genes from the first shell identified as most relevant to this input. The analysis has suggested several high-confidence modules, most of which contained prominent asthma-associated signalling pathways (*HIF* [29], *TGF-beta* [30] and cytokine–cytokine receptor interaction [31] and circadian clock-related [32]). Several high-degree nodes connecting these multiple modules are also known to be implicated in asthma: tyrosine kinase *FYN* is involved in inflammation [33], *IL1B* shown to be genetically associated with asthma susceptibility [34] and the node with the highest degree (*SMAD4*) is involved in airway smooth muscle hyperplasia [35].

Given that the number of identified human PPIs is now in the millions, visual exploration is typically limited by the sheer amounts of relevant information that can be usefully displayed. Approaches like network diffusion and graph random walks offer a mathematically robust way to select most relevant nodes even in large graphs. These methods are effective for disease analysis, as disease-related genes are commonly located close to each other in the network. In this example, we have demonstrated the applicability of this key principle and also identified 10 most relevant (closest to the signature) genes by the diffusion state distance metric [36]. This analysis highlighted potential relevance of the *PTEN* gene, known to be prominently involved in airway hyperresponsiveness and inflammation, [37] as well as a small module with multiple members of the *PI3K/Akt* pathway that promotes asthma-related airway remodelling [38].

## Contextualization by pathway centric representations

Understanding the mechanism of disease often starts with identification of the aberrant molecular pathways that may be associated with the disease. Molecular pathway models aim to capture sequences of actions or events of molecular ‘actors’. Some examples of commonly modelled events include biochemical reactions, state changes and movements between different cellular compartments. Mapping known disease genes to known pathways is a well-established methodology for contextualization, as pathways capture an intermediate level of organization between molecular entities and phenotype. The identification of hallmark pathways believed to be associated with disease conditions has been used to create pathway centric disease maps [39], which are useful for visual exploration and for the overlay of data from, for example gene expression studies. Typically, such approaches involved identification of disease-related pathways by careful manual curation of the literature. AlzPathway [40] is a collection of signalling pathways in Alzheimer’s disease. The map is represented in the Systems Biology Graphical Notation (SBGN) [41] process description (PD) language and was also made available in other formats. The resource also offers a Web interface that allows users to review and comment on annotations thus facilitating community curation. Another example of a pathway-based disease map is the Parkinson’s disease map [42], which builds on an understanding of the hallmark metabolic, regulatory and signalling pathways of the disease [39]. The Parkinson’s disease map can be accessed using the Minerva Web server, which has advanced functionality for visualization (using the GoogleMaps API) that facilitates manual exploration [43]. The Atlas of Cancer Signalling Networks [44] uses a similar approach, where several types of biological entities, like phenotype, ion and drug, can be represented on a zoomable map in SGBN format. Clicking on an entity brings up a box with its description and relevant annotations.

Ideally, a computational framework for contextualizing disease-implicated genes should allow annotation by members of the research community, and some selected frameworks offer this important functionality. WikiPathways [45] contains a set of open, evolving, updateable disease-associated pathways. PathWhiz [46] is a Web-based tool that allows users to draw pathways so as to include contextual information such as cells, tissues and organs. The pathway maps are designed to be visually appealing to facilitate manual exploration. PathWhiz includes metabolic, signalling, as well as a number of disease-associated pathways. Recently, the PathWay Collage tool [47] has been developed that allows the visualization of fragments of pathways (defined by the user so as to reduce the complexity of full pathway representations) onto which omics data can be overlaid.

At present, there are two leading exchange standards adopted for representing biochemical pathway networks. The first one is Systems Biology Markup Language (SBML) [48], which was primarily intended to support development and sharing of quantitative biochemical models. Therefore, SBML has been designed to be generic, with the main building blocks being species (quantifiable physical entities), processes (which define how entities are manipulated) and compartments (provide low-level context for processes and entities). As SBML format aims to be generic, further subcategories of these concepts are not defined as part of the standard, and instead, particular emphasis is placed on enabling mathematical definition of the model (e.g. in the form of ordinary differential equations). Some of the necessary biological underpinnings are added via a related project, SBGN [41] standard, which defines how different SBML entities can be represented graphically. In SBML fine-grained annotation or specification of complex contexts is possible through special fields set aside for user data. However, as these fields are not bound by the standard, they may not be supported by all of the tools and therefore generally would not be used in evaluation of the model.

The SBGN PD language [41] was designed to give an unambiguous representation of mechanism of action within a biological pathway that can be interpreted visually to facilitate manual exploration. The PD language can represent all the steps in, for example, a reaction showing how a biological entity changes in a temporal manner. However, to fully exploit the PD representation for applications like numerical modelling would require additional data associated with component steps (such as rate constants), which often is not readily available.

Another prominent data exchange standard is BioPAX [49], which was primarily designed for unambiguous sharing of biochemical pathway data and therefore offers much wider selection of domain-specific terms to characterize and categorize both interactors and interactions. As Ontology Web Language (OWL) forms the core of this standard, all of the terms are organized hierarchically and relationships between them are formally defined. BioPAX standard aims to facilitate data sharing and integration via Semantic Web technologies and Resource Description Format (RDF). If an RDF representation is used, the format can be easily extended to incorporate complex context information; however, BioPAX currently does not aim to offer the means of defining experimental or biomedical context for pathways, as, by design, other standards/ontologies can be brought in to model them.

Both SMBL and BioPAX are predicated on the developments of the past decade, when several key technologies and design principles were introduced that greatly influenced how biomedical data-sharing standards are implemented. One of these developments was increasing adoption of formal ontologies that allowed development of well-structured controlled vocabularies for different domains and definition of cross-mappings between them. For example, the SBML standard allows annotation of models with System Biology Ontology (SBO) terms, which are also used in SBGN and can be mapped on to BioPax via SBPAX [50]. Therefore, in principle, it is possible to integrate models across all of these standards on a qualitative level. On a syntactic level, different formalisms can be integrated by introducing support for a low-level common language, like RDF, which is representation-agnostic and allows all types of data to be linked via common identifiers. Both of these solutions enable modern standards to be designed in a modular way, where different specialized representations can be developed by different communities of domain experts and then combined together—or even extended for the needs of a particular applied project.

In recent years, the community efforts have been increasingly devoted to increasing inter-compatibility across all main biomedical standards. These efforts include development of converters, controlled vocabulary mappings and better support of different standards both by data providers and analytical tool developers. Greater compatibly also means that smaller models (e.g. from individual studies) can now be meaningfully shared and combined. One prominent resource that aims to facilitate this process is NDex [51]. NDex allows researchers to share their models in a variety of different formats, which can then be used directly by using the NDex website like a data warehouse or integrated into a common representation by using the NDex Cytoscape [52] plug-in.

**Figure 3:**
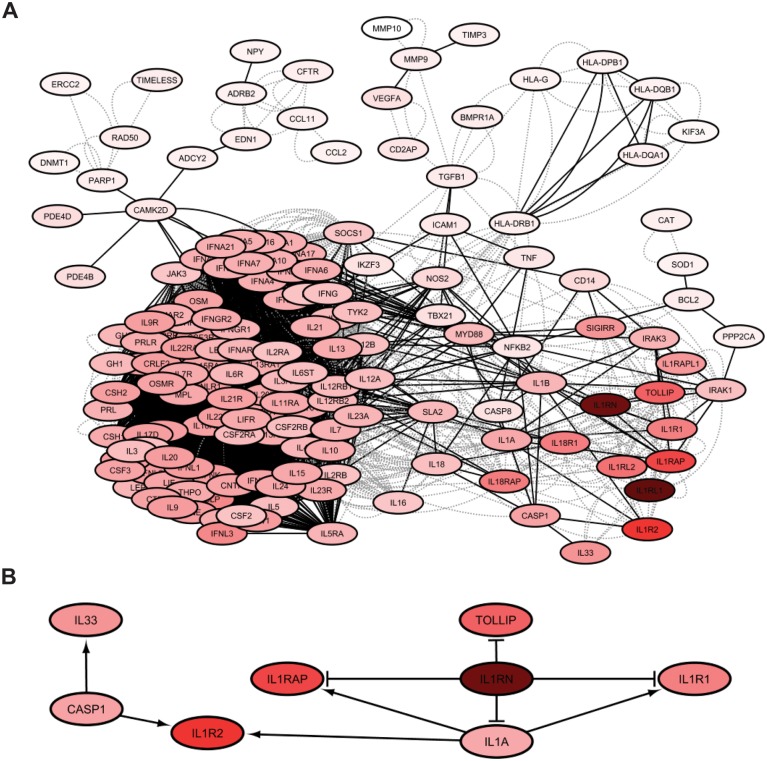
Significant pathways identified using Tied Diffusion through Interacting Events method, an approach that can extract the most likely set of interconnectors between two sets of nodes. In this example, core asthma genes from DisGeNET and severe differential expression signature were considered in the context of Reactome regulatory pathway network. In both panels, colour intensity indicates weight magnitude assigned by the algorithm. (**A**) Complete set of all significant genes. Dashed lines indicate undirected edges of ‘component’ type, whereas solid edges show directed interactions; (**B**) subset of the network relating to the *IL1RN* gene. Arrow and bar-terminated edge styles show activation and inhibition, respectively.

Pathway-based representations enable relevant methods to incorporate the directionality and interaction type (e.g. up- or down-regulation), and in this way, they can capture causal associations underlying different disease mechanisms. To illustrate this application, we have used ‘Tied Diffusion through Interacting Events’ method [53], which can identify significantly implicated pathways connecting two sets of genes (in this case DisGeNET asthma signature and our severe asthma signature). The diffusion model used by this method can take into account magnitude of effects, direction and type of interactions. After applying this approach, the regulatory subnetwork from Reactome found to be significant for these two sets was visualized in Cytoscape (Figure 3). It is possible to see that two interleukin genes (both involved airway inflammation [54]) were found to be particularly critical, with lower panel showing key associations involved.

## Contextualization by knowledge graph representations

There are many specialist sources of disease-related information such as databases of mutations and their consequences. In addition, there is vast information in unstructured formats in the scientific literature and much of it is contextual, for example statements that describe correlations between gene products or between small molecules and gene products that are observed in particular cell types. Information in the literature may also describe weak associations only seen in certain conditions, or for which there may be limited evidence that could nevertheless still be useful for hypothesis generation.

The Biological Expression Language (BEL) [55] represents statements in biology, typically correlative and causative statements as well as the context in which these statements apply. A biological statement is represented as a triple (subject, predicate and object) where the subject is a BEL term and the predicate is a biological relationship that connects the subject with object. A BEL term can be the abundance of a biological entity or it can be a biological process such as one described in the Gene Ontology (GO) [56], or even a given disease condition. Each entity is described in an existing namespace (e.g. from databases like ChEBI [57]) or in particular user-defined namespace(s). There is a fixed set of causal, correlative and other relationships that link these entities. The BEL complier checks that the statements are syntactically correct and carries out the integration and alignment of the data by establishing equivalences between identical concepts.

The knowledge captured by BEL statements can be represented as a graph. The object can be a BEL term or a BEL statement. Each statement can be associated with metadata that contextualizes it, for example qualifying it to be true only in specific tissues. The BEL knowledge network can facilitate data interpretation using a reverse causal reasoning approach [58]. For example, gene expression data can be mapped to smaller subnetworks that represent cause and effect models. The extent to which the models can explain the measurements of differentially expressed genes can then be assessed and potentially yield insight into the mechanism.

Hofmann-Apitius [5] posits that an understanding of disease mechanisms involves integrating information at multiple biological scales. To some extent, BEL offers a multiscale representation that other languages do not. Using BEL, Hofmann-Apitius and co-workers have explored mechanistic aspects of Alzheimer’s disease [59–62]. They used the BEL language to represent information from the literature together with relevant experimental data sets to build integrated evidence networks. This approach helps to mine what they refer to as the knowledge ‘grey zone’, which includes both established and emerging connections between entities. This approach can be powerful for linking mechanisms associated with biomarkers [60].

Constructing such information-rich representations can involve considerable manual curation efforts. For this reason, TM will be increasingly important for supporting this process [63]. Already existing examples include the BEL Information Extraction workFlow (BELIEF) system and BELSmile [64]. These frameworks work by using automated TM to detect BEL concepts and relationships and then allowing the curators to explore and annotate their biological contexts to create final high-quality networks.

Another example of a specialized curation data modelling format is the Nanopublication standard [65]. Nanopublication defines rules for publishing data in its simplest possible forms—individual assertions in the form of statements composed of a subject, predicate and object, plus supporting provenance information and relevant metadata. Nanopublication is designed to be used in combination with Semantic Web technologies and uses RDF, a format that allows seamless incorporation of ontology terms and cross-references through globally unique shared identifiers. The advantages of this standard include decentralized (federated) publishing, machine-readable representation of data and an ability to track provenance at the highest possible level of granularity.

Knowledge network representations of biological data are increasingly being leveraged in in integrative analysis platforms, which aim to combine information resources with common analysis and visualization tasks. One example of such platforms is Ingenuity Pathway Analysis (IPA^®^). IPA^®^ suite aims to facilitate interpretation of experimental data from high-throughput omics experiments and includes methods for identification of causal networks and visual exploration of biological graphs [66]. The BioMax BioXM™ platform uses an object relational database architecture to store, query and envision disease information, including molecular, genetic, physiological and clinical data, as well as biological models. This platform has been used to create a resource for chronic obstructive pulmonary disease (COPD) called ‘COPD knowledge base’ [67–70]. The authors of COPD knowledge base do acknowledge that much disease-related information remains hidden in the literature and also recognize the difficulties of capturing quality and context specificity. The Malacards resource [71] uses a relational database for the semantic integration of multiple sources of information about diseases, and also provides network visualizations of disease–disease relationships. GeneAnalytics [72] enables contextualization of functional signatures (gene sets) in the context of organs and tissues and compartments, as well as other annotation sources, building on the GeneCards [73] and Malacards technologies.

Recent advances in graph-based databases have opened up additional possibilities for organizing heterogeneous and interlinked data. Graph databases have several features that make them particularly attractive for management and exploratory analysis of complex biological data sets. A particularly noteworthy feature is excellent performance of traversal-type queries across entities of diverse types they offer. Such queries could be challenging to realize in relational databases because of the cost of computing statements across different tables. Traversal queries can be an important way of identifying new relationships in integrated data, particularly in the cases where links only become apparent once multiple sources have been integrated, but may not be immediately obvious because of sheer amounts of data being involved. Modern graph databases offer sophisticated query languages and often also other types of framework-specific functionality to facilitate applied analysis, like an ability to extend the framework with user-defined functions [74] and integration with ontologies and reasoning systems. One example of a graph database solution that has been already used in biomedical domain [75–78] is Neo4J DBMS. Neo4J is a Java-based solution, which also offers its own graph query language (Cypher), which is conceptually similar to SQL.

Semantic Web [79], RDF [80] and Linked Open Data [81] are emerging important standards for sharing biomedical data. At its core, Semantic Web is characterized by its use of globally unique, unambiguous identifiers for entities. The identifier used for this purpose is commonly a web link (URL). The RDF format is structured as a set of statements with three parts (subject, predicate and object), where a subject and object are entities with a unique identifier, and predicate defines the type of a relationship between them. Linked Open Data standard established a set of rules for providing meaningful RDF at the resolvable identifier URL, which generally is in the form of statements that provide meaningful information about that entity that can in turn link it to other URL-identified entities, and, finally, the entities and statements can be linked to specialized ontologies in OWL [82] that provide additional information about how to interpret them within a particular context.

From the perspective of biomedical data integration, RDF primarily plays the role of a low-level data exchange standard useful for addressing the syntactic heterogeneity issue and facilitating data integration. Although RDF representations can be interpreted as integrated knowledge networks, usually an additional set of standards will be necessary for meaningful interpretation of data from a biological perspective. Prominent resources that offer biological data on the Semantic Web include EBI RDF platform [83] (covers such important databases like UniProt [84], Reactome [85], Ensembl [86], ChEMBL [87] and Expression Atlas [88]), Bio2RDF (aims to mirror in RDF most of the prominent biological databases) [89] and OpenPhacts (uses Semantic Web technologies to represent the chemogenomics space) [90]. Some noteworthy ontologies used in combination with these resources include Experimental Factor Ontology (EFO) [91], SBO [92], Translational Medicine Ontology (TMO) [93], SNOMED (medical terms and concepts) [94] and GO (functional annotation of genes and proteins) [56].

**Figure 4:**
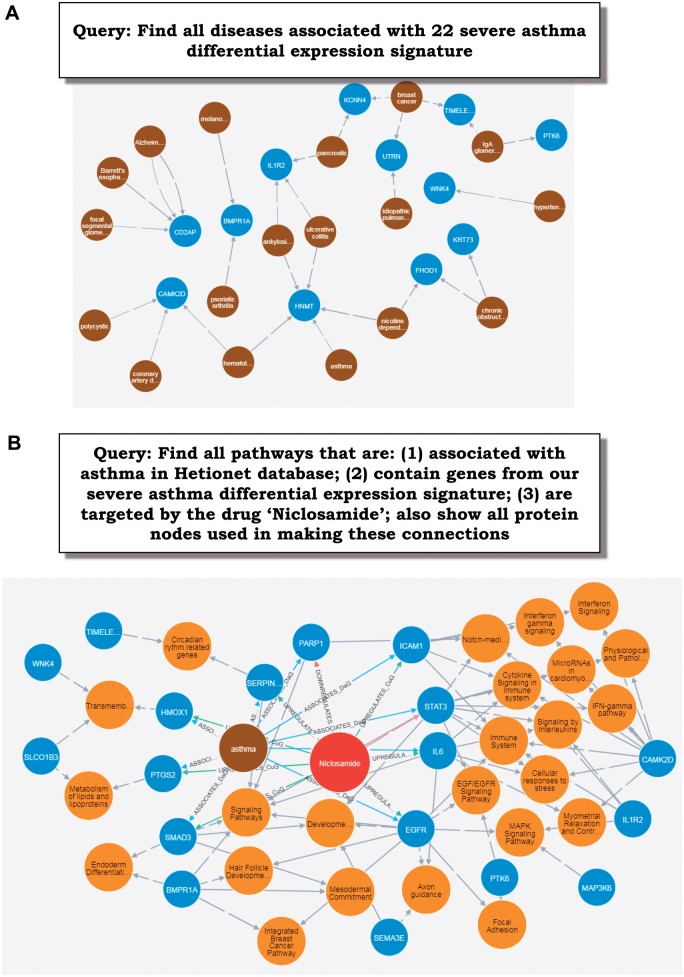
Query expressed in natural language and resulting output produced for the differential expression gene signature using Hetionet graph database. The following entities are shown: proteins (blue), pathways (yellow), diseases (brown) and drugs (red). (**A**) Query for associated diseases; (**B**) query to explore the connections between niclosamide drug, asthma and differential expression signature linked via relevant proteins and pathways.

To illustrate the application of integrated knowledge networks with our example 22-gene severe asthma signature, we have used a Neo4j-based Hetionet v1.0 resource [95] that integrates a wide variety of biomedical information. First, a query was prepared to visualize all of the diseases linked to the signature genes (Figure 4). According to these results, only one gene has already been linked to asthma, though three other genes have been linked to other lung diseases (COPD and idiopathic pulmonary fibrosis). Next, the connections between known asthma-related pathways, severe asthma differential expression signature and drugs targeting those common pathways were retrieved. In terms of query-specific degree of drug nodes (i.e. number of links to returned proteins), top three drugs were dexamethasone, betamethasone and niclosamide. The former two are corticosteroids already used for treating asthma [96], and the last one was recently proposed as a highly promising treatment because of its strong *TMEM16A* antagonism found to improve lung function in human and mouse models [97].

## Approaches for extraction of contextual knowledge

Ultimately, all types of models in biomedical domain originate from data collected during relevant experiments, and such data would typically require both different levels of preprocessing and interpretation to yield such models. At present, relatively few types of raw experimental data can be straightforwardly converted into these representations without requiring human interpretation. Some possible examples are construction of co-expression networks [98, 99] from transcriptomic data and sequence homology graphs [100, 101]. However, currently, manual interpretation and publishing of inferred models as free text in scientific papers remains the primary means of disseminating this knowledge, and all major biomedical databases discussed in this review expend considerable curation efforts to collect, verify and consolidate it in standardized formats from source literature.

Given the volume and ever-increasing rate of scientific publishing, automated means of facilitating this process are becoming increasingly essential. This is achieved through applications of information retrieval (IR) and TM methods, which can handle tasks like identification of relevant documents, sections and sentences, recognition of concepts and relationships between them or even generation of novel hypotheses [102]. Introduction of computational text analysis methods into the curation process has been reported to improve its efficiency by between 2- and 10-fold [103]. Especially in the biomedical community, development of TM approaches has greatly benefited from competitive evaluation challenges [104]. Some prominent examples of such initiatives include BioCreative, TREC Genomics/Medical/CDS, BioNLP-ST, i2b2 and ShARe/CLEF eHealth. These regular competitions provide a valuable benchmark for current state of the art for different tasks and serve to highlight the most important problems, focus community efforts and establish necessary annotated textual resources.

As well as assisting with curation, both TM and IR can be applied in a high-throughput and automated manner; however, the utility of these applications is still limited by accuracy and generalizability [103]. In its simplest form, various statistical co-occurrence methods can be applied to build networks linking biological concepts frequently found together in different papers. Some examples of resources offering such literature-derived data are miRCancer that compiled data about co-occurrence of microRNA mentions with different cancer types [105] and DISEASES resource, which provides similar information for gene mentions with particular diseases [106].

Extraction of information from text is contingent on unambiguous identification of entities and relationship types from which model statements can be constructed. Therefore, TM technologies have a natural synergy with ontology-based data modelling, and to a lesser extent Semantic Web formats, like RDF [102, 107]. The ontologies are used both as controlled vocabularies to resolve alternative naming conventions and as sources of common identifiers for recovered concepts and relationships. Some examples of specialized relationship types that can be recovered include protein–protein [108], drug–protein [109] and drug–drug [110, 111] interactions, and associations of genes with mutations [112, 113] and phenotypes [114–116]. Data from IR and TM are increasingly incorporated alongside manually reviewed information by major databases, for example PPIs extracted from literature by fully automated approaches are offered by STRING [13, 117].

To handle more advanced tasks, it is usually necessary to combine specialized tools into TM workflows, e.g. a hypothesis generation tool might rely on output of a relationships identification tool, which in turn relies on named entity recognition. Inevitably, this raises questions of interoperability of different analysis methods. One way to address this is has been through increasing use of Web services to link up different tools [118]. Additionally, specialized standards for biomedical textual data interchange were developed, for example XML-based BioC format [119].

Recent efforts have been exploring the possibility of extracting more complex statements from text [120], and in 2015, the BioCreative V challenge has for the first time included a task of automated extraction of OpenBEL statements from text [121]. During the competition, the best result was achieved by the BELMiner framework [122], which used a rule-based approach for extraction of BEL statements from text and got the highest *F*-measure of 20% for this task. After the challenge, BELSmile system was developed that reached an even better score of 28% on the same dataset [64]. BELSmile implemented a semantic-role-labelling approach, which relies on recognition of verbs (predicates) and assignment of roles to associated subjects/objects relative to that verb. Notably, designs of both frameworks integrated multiple specialized IR and TM tools to handle specific low-level subtasks.

It is clear that at present a substantial proportion of valuable biomedical knowledge is ‘trapped’ in scientific text, in a representation not readily suitable for automated computer-driven interpretation. Appropriate data formats and ontologies are an essential prerequisite for enabling sophisticated TM methods to begin addressing this challenge. The most recent developments indicate that current TM approaches are becoming powerful enough to leverage such rich representations and are already useful to curators working on complex models of human diseases. To explore further developments in this important area, we direct reader to the following recent reviews [25, 102, 123].

## Outlook

Many translational and systems medicine projects involve collection of molecular and clinical data with a view to the identification of disease subtypes. The data are typically warehoused and analysed, for example, to extract molecular fingerprints that characterize the subtypes, and this data interpretation step of the translational informatics pipeline remains highly challenging. Moving from diagnostic molecular patterns to being able to suggest individualized healthcare pathways is complex. Data integration links the molecular patterns to background knowledge, which can then be reviewed, explored and analysed to develop a more detailed understanding of the underlying biology that distinguishes disease subtypes.

To support this process, several standards have emerged to capture, integrate and facilitate analysis of these data. This proliferation of standards suggests that different formalisms will continue to be necessary to model different aspects of biomedical data and that we are unlikely to converge on one ultimate modelling solution in the foreseeable future. The open question therefore becomes how to effectively manage the interoperability between these different representations? In this respect, several common trends and best practices are beginning to emerge. In particular, it is now evident that development of common controlled vocabularies and specialized domain ontologies is essential for effective management of increasingly complex biomedical data. Use of ontologies facilitates identification of equivalent entities and concepts across different modelling formats and facilitates conversion between them, allowing efficient data integration.

As outlined by Cohen in [6], modern biomedical data modelling formalisms can be categorized into ‘curation’ and ‘mechanistic’ types. Aim of the former is to primarily describe experimental results (e.g. Nanopublications, BioPAX), including observed associations between molecular signatures and diseases. The latter specify evaluable models used to simulate living systems and generate predictions based on a set of inputs (e.g. SBML, Kappa). It is obvious that although experimental observations are ultimately used to construct such models, the processing steps required to correctly generate the latter from the former would go beyond a simple format change. We believe that automation of this process is a highly promising emergent strategy for development of fully mechanistic models of disease. Use of automated methods to support biomedical innovation is necessitated both by the need to effectively deal with ever-growing volumes of data and by the increasingly complex nature of biomedical research itself. The apparent diminishing returns in terms of novel drugs successfully taken to market relative to the money spent [124, 125], with particular challenges for cardiovascular [126] and neurodegenerative diseases [127, 128], mean that increasingly more complex strategies for managing disease are needed to make progress [129]. Consequently, high-throughput computational approaches are becoming indispensable both for assisting with interpretation, management and mining of experimental data as well as for constructing and evaluating relevant clinical models. At present, cutting-edge efforts in this area involve creation of increasingly sophisticated automated methods for analysis of vast quantities biomedical data, exemplified by initiatives like DARPA’s Big Mechanism, IBM Watson Health and Garuda biomedical discovery platforms. The future modelling formats are therefore likely to be influenced by the requirements of such projects and will increasingly incorporate features to support fully computer-driven information analysis.

In our view, accurate handling of the context of biological observations is critical for construction of correct mechanistic models of disease. Traditionally, information has been compiled into largely homogenized collections like pathway maps or global interactome networks that aim to show all possible processes of a certain type for a given organism. However, in practice, only a small subset of all these processes will be occurring in a real cell at a given time. Furthermore, such events may be transient (enzymatic reactions), have stable outcome (protein complex formation) or be mutually exclusive (alternative protein-binding partners). An additional level of complexity is introduced from the imperfection of experimental techniques. For example, transcriptomics profiling is often done at a tissue level, which may mask important differences at a level of individual cells. As such, mechanistic models built from these data may be subject to considerable uncertainty and abstraction. The next generation of formats and integration solutions will therefore need to be more context aware. Future progress on one hand requires better means of expressing this highly granular context of biological processes and on the other necessitates solutions to model vast volumes of data and knowledge in such form. Once efficient and accurate approaches for these tasks are established, cutting-edge mathematical and machine learning methods could be leveraged to their full potential to give new insight into disease mechanisms and suggest improved avenues for therapeutic intervention.

###

**Key Points**

- Precision medicine relies on identification of disease subtypes at a molecular level and linking them to diagnostic models to determine optimal treatment strategies.
- In practice, establishing a contextual link between molecular signatures and disease processes requires integration of qualitatively diverse types of relevant data and knowledge.
- At present, several alternative philosophies guide the process of transformation of experimental data into mechanistically informative and ultimately, clinically actionable insights.
- The spectrum of possible approaches ranges from purely associative knowledge link discovery to fully quantitative mathematical models.

## Funding

The research leading to these results has received support from the Innovative Medicines Initiative Joint Undertaking eTRIKS Project (Grant Number: IMI 115446) resources of which are composed of a financial contribution from the European Union's Seventh Framework Programme (FP7/2007-2013) and European Federation of Pharmaceutical Industries and Associations (EFPIA) companies’ in kind contributions. The work was also partially supported by Core Research for Evolutional Science and Technology (CREST) Grant from the Japan Science and Technology Agency (Grant Number: JPMJCR1412), and Japan Society for the Promotion of Science KAKENHI (Grant Numbers: 17H06307 and 17H06299).

## Supplementary Material

bby025_Supp

## Notes

**Mansoor Saqi** is a Research Fellow at the Data Science Institute at Imperial College London. His research interests are in translational medicine informatics, in particular data integration, and analysis of biological networks.

## Notes

**Artem Lysenko** is a Postdoctoral Researcher at RIKEN Laboratory for Medical Science Mathematics. His research is in the areas of biomedical machine learning, applied data science and biological network analysis.

## Notes

**Yi-Ke Guo** is the Director of the Data Science Institute at Imperial College London. He has been working on technology and platforms for scientific data analysis since the mid-1990s. His research focuses on knowledge discovery, data mining and large-scale data management. He is a Senior Member of the IEEE and a Fellow of the British Computer Society.

## Notes

**Tatsuhiko Tsunoda** is a Full Professor at Tokyo Medical and Dental University, and a Chief Scientist and the Director of Laboratory for Medical Science Mathematics at RIKEN. His research interest is in development and applications of mathematical models and algorithms for genome analysis, multiomics and precision medicine.

## Notes

**Charles Auffray** is the Founding Director of the European Institute for Systems Biology and Medicine (EISBM) Lyon. He develops a systems approach to complex diseases, integrating functional genomics, mathematical, physical and computational concepts and tools. He has participated in or coordinated multiple European Union projects.
