# Supplementary material for: Navigating the disease landscape: knowledge representations for contextualizing molecular signatures
Source: Brief Bioinform. 2018 Apr 19;20(2):609–23. doi: 10.1093/bib/bby025 (PMC6556902; doi:10.1093/bib/bby025)
Supplement: bby025_Supp [file bby025_supp.docx]

**Supplementary Methods**

**Identification of asthma-related gene signatures**

Processed expression matrix was for GSE43696 study was downloaded from the GEO database [[1](#_ENREF_1)]. Differential expression was performed using Empirical Bayes method [[2](#_ENREF_2)] (from R edgeR library) on the log-transformed probeset-level data, using the sample sets identified as mild and severe asthma by the original authors of the study. Significance values were corrected for multiple testing using FDR method and final probesets were chosen at the p<0.05 significance level cutoff. In cases where multiple probesets were resolved to the same gene, the set with highest absolute fold-change was chosen. This resulted in a 22-gene differential expression signature.

For the second signature, manually curated genes associated with asthma were taken from DisGeNET database [[3](#_ENREF_3)]. These annotations were found to be predominantly based on evidence of genetic association with asthma curated from relevant papers. This set had 99 genes, each assigned a particular evidence strength score by the source database.

**Protein-protein interactions and diffusion state distance computation**

Gene identifiers from expression signature were used to query STRING database [[4](#_ENREF_4)] via multi-gene web interface. To control for false positives, a confidence cut-off of 0.2 was set to filter the edges. The main image was generated by selecting 50-gene 1^st^ level shell. Then, functional annotation interface of STRING was used to explore common function of modules and key genes.

For diffusion state distance analysis, complete STRING database for human was downloaded and filtered to retain only experimentally-verified edges with combined score above 200 and genes in the main connected component of the resulting graph. Gene identifiers were mapped to STRING nodes using STRINGdb R package. This resulted in 20 genes in differential expression signature and 97 genes in DisGeNET signature. The diffusion state distance was calculated as described in original paper by [[5](#_ENREF_5)], using the following formula:

$$DSD\left( u,v \right)= \left\| {({x_{u}}^{T}-{x_{v}}^{T})(I-D^{-1}A+P)}^{-1} \right\|_{1}$$

Where *u* and *v* are a pair of nodes, *P* is the constant matrix of the steady-state distribution, *x* is the relevant basis vector and *D* - a diagonal degree matrix. Profiling of distances between members of different signatures was done by considering all pairwise distances of respective members. A random subset was generated by drawing a set of 1000 random node pairs from the main component of the network.

**Identification of significantly implicated pathways**

To explore the pathway-specific context of the signature genes, they were mapped to the Functional Interactions network downloaded from the Reactome database. Signature data was processed to make it conformant with the input of the TieDIE method [[6](#_ENREF_6)]. Specifically, DisGeNET signature was chosen as an upstream set, with DisGeNET score as weight and all signs set to positive and differential expression signature was chosen as a downstream set with absolute log-fold change value as weight and sign taken from direction of fold-change.

TieDIE method uses bi-directional diffusion that respects both logical implication of the effect and direction of the edges in the network. For performance reasons, heat diffusion kernel matrix used by the method was pre-computed in R according to the formula [[6](#_ENREF_6)]:

$$K\left( A \right)=e^{-\left( L \right)t}$$

Where *L* is the Laplacian matrix for corresponding adjacency matrix *A* and *t* parameter was set to the recommend value of 0.1. A significant subset of pathways was computed using 1,000 permutations (resulting in p-value of 0.0029). Full subset of significant pathway links and nodes heat file were imported into Cytoscape; IL1RN sub-network was selected by filtering to remove “component” edges and nodes with heat value below 0.3.

**Extraction of disease and candidate drug information**

Additional annotation was extracted via Neo4j front-end of the Hetionet database (<https://neo4j.het.io>) [[7](#_ENREF_7)] and executing three relevant queries in Cypher language. To extract all diseases linked to signature genes:

**MATCH (d1:Disease)-[r1]-(g1:Gene)**

**WHERE g1.name IN ['WNK4', 'SLCO1B3', 'KCNN4', 'CPXM1', 'SYT13', 'KRT73', 'SEMA3E', 'CD2AP', 'IL20RB', 'NAT8B', 'AATBC', 'BMPR1A', 'CAMK2D', 'FHOD1', 'MYO19', 'PTK6', 'CHL1-AS1', 'TIMELESS', 'DPPA5', 'UTRN', 'MAP3K6', 'HSD17B13', 'HNMT', 'IL1R2']**

**RETURN ***

For the second part, two queries were executed. The first query extracted counts of connections (links to proteins) for drugs connected to pathways common to our severe asthma differential expression signature and proteins associated with asthma in Hetionet:

**MATCH (d1:Disease)-[r1]-(g1:Gene)-[r2]-(p1:Pathway)-[r3]-(g2:Gene), (g1)-[r4]-(c1:Compound)**

**WHERE d1.name='asthma' AND g2.name IN ['WNK4', 'SLCO1B3', 'KCNN4', 'CPXM1', 'SYT13', 'KRT73', 'SEMA3E', 'CD2AP', 'IL20RB', 'NAT8B', 'AATBC', 'BMPR1A', 'CAMK2D', 'FHOD1', 'MYO19', 'PTK6', 'CHL1-AS1', 'TIMELESS', 'DPPA5', 'UTRN', 'MAP3K6', 'HSD17B13', 'HNMT', 'IL1R2']**

**RETURN c1.name, COUNT(r4)**

**ORDER BY COUNT(r4) DESC**

Then, based on this information, follow-up query was constructed to display information relevant to the third-highest drug by query-specific degree, Niclosamide:

**MATCH (d1:Disease)-[r1]-(g1:Gene)-[r2]-(p1:Pathway)-[r3]-(g2:Gene), (g1)-[r4]-(c1:Compound)**

**WHERE d1.name='asthma' AND g2.name IN ['WNK4', 'SLCO1B3', 'KCNN4', 'CPXM1', 'SYT13', 'KRT73', 'SEMA3E', 'CD2AP', 'IL20RB', 'NAT8B', 'AATBC', 'BMPR1A', 'CAMK2D', 'FHOD1', 'MYO19', 'PTK6', 'CHL1-AS1', 'TIMELESS', 'DPPA5', 'UTRN', 'MAP3K6', 'HSD17B13', 'HNMT', 'IL1R2'] AND c1.name IN ['Niclosamide']**

**RETURN ***

Results of both queries were visualised using default capabilities of web-based Neo4j client.

**References**

1. Edgar R, Domrachev M, Lash AE. Gene Expression Omnibus: NCBI gene expression and hybridization array data repository, Nucleic acids research 2002;30:207-210.

2. Smyth GK. Linear models and empirical bayes methods for assessing differential expression in microarray experiments, Statistical applications in genetics and molecular biology;3:1-25.

3. Piñero J, Queralt-Rosinach N, Bravo À et al. DisGeNET: a discovery platform for the dynamical exploration of human diseases and their genes, Database 2015;2015.

4. Szklarczyk D, Franceschini A, Wyder S et al. STRING v10: protein–protein interaction networks, integrated over the tree of life, Nucleic acids research 2014;43:D447-D452.

5. Cao M, Zhang H, Park J et al. Going the distance for protein function prediction: a new distance metric for protein interaction networks, PloS one 2013;8:e76339.

6. Paull EO, Carlin DE, Niepel M et al. Discovering causal pathways linking genomic events to transcriptional states using Tied Diffusion Through Interacting Events (TieDIE), Bioinformatics 2013;29:2757-2764.

7. Himmelstein DS, Lizee A, Hessler C et al. Systematic integration of biomedical knowledge prioritizes drugs for repurposing, Elife 2017;6.
